# *In vitro* method for 3D morphometry of human articular cartilage chondrons based on micro-computed tomography

**DOI:** 10.1016/j.joca.2018.05.012

**Published:** 2018-08

**Authors:** I. Kestilä, J. Thevenot, M.A. Finnilä, S.S. Karhula, I. Hadjab, S. Kauppinen, M. Garon, E. Quenneville, M. Haapea, L. Rieppo, K.P. Pritzker, M.D. Buschmann, H.J. Nieminen, S. Saarakkala

**Affiliations:** † Research Unit of Medical Imaging, Physics and Technology, Faculty of Medicine, University of Oulu, Oulu, Finland; ‡ Infotech Oulu, University of Oulu, Finland; § Department of Applied Physics, University of Eastern Finland, Kuopio, Finland; ‖ Medical Research Center, University of Oulu, Oulu, Finland; ¶ Institute of Biomedical Engineering, Ecole Polytechnique de Montreal, P.O. Box 6079, Station Centre-Ville, Montreal, Quebec H3C 3A7, Canada; # Biomomentum Inc., 970 Michelin St., Suite 200, Laval, Quebec H7L 5C1, Canada; †† Department of Diagnostic Radiology, Oulu University Hospital, Oulu, Finland; ‡‡ Department of Laboratory Medicine and Pathobiology, University of Toronto, Toronto, Ontario, Canada; §§ Mount Sinai Hospital, Toronto, Ontario, Canada; ‖‖ Groupe de Recherche en Sciences et Technologies Biomédicales, Polytechnique Montreal, P.O. Box 6079, Station Centre-Ville, Montreal, Quebec H3C 3A7, Canada; ¶¶ Department of Physics, University of Helsinki, Helsinki, Finland; ## Department of Neuroscience and Biomedical Engineering, Aalto University, Espoo, Finland

**Keywords:** Hexamethyldisilazane, Osteoarthritis, Segmentation, Morphology, 3D analysis

## Abstract

**Objective:**

The aims of this study were: to 1) develop a novel sample processing protocol to visualize human articular cartilage (AC) chondrons using micro-computed tomography (μCT), 2) develop and validate an algorithm to quantify the chondron morphology in 3D, and 3) compare the differences in chondron morphology between intact and osteoarthritic AC.

**Method:**

The developed protocol is based on the dehydration of samples with hexamethyldisilazane (HMDS), followed by imaging with a desktop μCT. Chondron density and depth, as well as volume and sphericity, were calculated in 3D with a custom-made and validated algorithm employing semi-automatic chondron selection and segmentation. The quantitative parameters were analyzed at three AC depth zones (zone 1: 0–10%; zone 2: 10–40%; zone 3: 40–100%) and grouped by the OARSI histological grades (OARSI grades 0–1.0, *n* = 6; OARSI grades 3.0–3.5, *n* = 6).

**Results:**

After semi-automatic chondron selection and segmentation, 1510 chondrons were approved for 3D morphometric analyses. The chondrons especially in the deeper tissue (zones 2 and 3) were significantly larger (*P* < 0.001) and less spherical (*P* < 0.001), respectively, in the OARSI grade 3–3.5 group compared to the OARSI grade 0–1.0 group. No statistically significant difference in chondron density between the OARSI grade groups was observed at different depths.

**Conclusion:**

We have developed a novel sample processing protocol for chondron imaging in 3D, as well as a high-throughput algorithm to semi-automatically quantify chondron/chondrocyte 3D morphology in AC. Our results also suggest that 3D chondron morphology is affected by the progression of osteoarthritis (OA).

## Introduction

With osteoarthritis (OA), chondrocytes are known to become hypertrophic and to form clusters, indicating that they may play a critical role in the increased metabolism observed in this condition^1^, ^2^. The mechanisms that initiate these changes, however, are not fully understood. One explanation is that the chondrocytes produce more extracellular matrix (ECM) macromolecules trying to compensate the increased cartilage degeneration, which then leads to increased cellular clustering and hypertrophy^1^. Previous studies have also shown that not only the anabolic factors, but also the catabolic factors that contribute to ECM degradation are expressed in OA chondrocytes^3^, ^4^. At the level of the chondron – the chondrocyte and its complex pericellular microenvironment^5^, ^6^, ^7^ – previous studies have suggested that the cleavage of fibrillary collagens initiates chondron enlargement, which continues due to pericellular matrix deposition^8^, ^9^. Both the chondrocyte and its pericellular matrix (PCM) thus appear to be modulated by OA^8^, ^10^. While combining chondrocyte/chondron morphology with pathological pathways may yield new cues for understanding OA and cartilage metabolism, a lack of high-throughput tools currently exist for 3D chondron morphometry.

Some of the currently available methods for the quantification of 3D chondrocyte/chondron morphology include serial sectioning^11^, ^12^, confocal^13^ and multiphoton microscopy^14^, and synchrotron-based micro-computed tomography (μCT)^15^. Serial sectioning is a method in which multiple consecutive sections are cut from one sample and imaged; a 3D image is then reconstructed from the collected images^16^. The main problem with this method – besides the destruction of the sample – is that the cutting could damage the articular cartilage (AC) structures, including those of chondrons. While optical methods such as confocal and multiphoton microscopy^17^, ^18^, can provide 3D images without destroying the specimen, their drawbacks are the limited light penetration in opaque tissues and the spherical aberration that results from refractive index mismatch^19^. It has been shown that synchrotron-based μCT can provide 3D information from chondrocytes^15^, although limited access to synchrotron facilities restricts the use of this approach. Other modalities, such as focused ion beam scanning electron microscopy and transmission electron microscopy, are also available for tissue 3D imaging within limited sample sizes^15^.

Different μCT techniques are capable of providing volumetric information from the bone microarchitecture and cartilage structure of osteochondral samples^20^, ^21^. Conventional desktop μCT imaging of AC requires contrast agents to provide distinguishable contrast between the structures^22^, ^23^, ^24^, ^25^. However, current contrast enhanced μCT (CEμCT) methods are based on the contrast agent distribution in the tissue, which is affected by the electrostatic repulsion or attraction between the tissue constituents and the contrast agent molecule. Thus, the CEμCT can only provide indirect information about the composition of the AC, which may limit its use as a quantitative tool for morphometric chondrocyte analysis.

When imaging dried samples, the contrast arises from the tissue itself, not from external contrast agent distribution. And because native chondrocytes contain mostly water, which is removed during the drying process, the contrast between the chondrons and the ECM is enhanced. Hexamethyldisilazane (HMDS)-based air-drying was first proposed in 1983 as an alternative method for critical-point drying^26^. The surface detail in insect tissues and cells has been shown to be well maintained in dried HMDS-treated specimens^26^, ^27^, ^28^, ^29^. Because HMDS has lower surface tension than water and is able to cross-link proteins, the sample will likely not fracture and collapse during the drying process^26^, ^29^. HMDS drying has never been used in μCT cartilage imaging, however.

In this study, we present a novel HMDS-based sample processing protocol to enable *in vitro* chondron imaging from human AC samples using desktop μCT. The objectives were to develop a new sample processing protocol for μCT imaging and a semi-automatic algorithm to select and segment chondrons in 3D. The developed methodology was applied to human osteochondral samples in order to compare the chondron morphology within intact and osteoarthritic AC at different tissue depths.

## Method

### Sample preparation

**Fig. 1:**
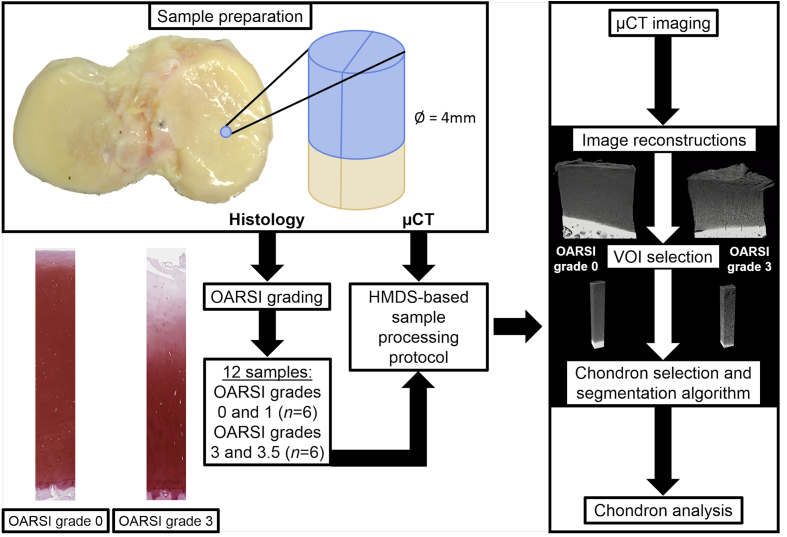
The pipeline of the methodology. First, the osteochondral cores were prepared from the tibiae and cut in half. The first half was subjected to histology (OARSI grading), and the second half to μCT. Based on the OARSI grades, 12 samples were selected for this study: six samples with OARSI grades 0–1.0, and six samples with OARSI grades 3–3.5. After the HMDS-based sample processing protocol, the samples were imaged with the μCT device, followed by image reconstructions and VOI selection. The chondron selection and segmentation algorithm was then applied to the VOIs, and finally, chondron analysis were performed.

Tibial plateaus of two cadaveric human donors asymptomatic of cartilage-related diseases (ages 26 and 49, body mass indices (BMIs) 18.4 and 30.6, one male and one female; RTI Surgical tissue bank, FL, USA) and four patients who had undergone total knee replacement surgery (ages 51–67, mean BMI 29.8, two males and two females; Maisonneuve-Rosemont Hospital, Montreal, Canada) were used in this study. The study was conducted under institutionally approved ethic committee certificates (CÉR-13/14-30 for Polytechnique Montreal and CÉR 14060 for Maisonneuve-Rosemont Hospital). The tibiae were preserved at −80°C, thawed once, transported at −20 to 0°C, and preserved again at −80°C before thawing for sample preparation. Osteochondral cores (diameter 4 mm) were prepared from both medial and lateral sides of the tibial plateaus and cut in half. One half was subjected to histological analysis and the other to HDMS-based μCT imaging. A pipeline figure visualizing the workflow of this study is shown in Fig. 1.

### Histological analyses

Osteoarthritis Research Society International (OARSI) histological grading^30^ was used to evaluate the OA progression of the samples (first half of the osteochondral core). The grading was performed for three consecutive 3-μm-thick Safranin O–stained histological sections. Sections were imaged with a virtual light microscope (Aperio AT2, Leica Biosystems, Wetzlar, Germany) using 40× magnification and 0.25 μm pixel size. The OARSI grading was first performed independently by three graders [SSK, LR, IK; inter-observer reliability: intraclass correlation coefficient (ICC) = 0.93, 95% confidence interval (CI) = (0.84; 0.98)], and their consensus grade was given as a final grade for each sample. Finally, twelve samples were selected into two groups: intact cartilage (*n* = 6, OARSI grades 0 and 1) and degenerated cartilage (*n* = 6, OARSI grades 3 and 3.5).

### HMDS-based μCT imaging

After preparation, the μCT samples (second half of the osteochondral block) were fixed in 4% saline-buffered formaldehyde for at least 5 days. Fixed samples were then dehydrated in ascending ethanol concentrations (30%–50%–70%–80%–90%–96%–100%) for a minimum of 3 h in each step, treated with HMDS for 3 h, and finally air-dried in a fume hood at room temperature overnight (details in the Supplementary material). A desktop μCT (SkyScan 1272, Bruker microCT, Kontich, Belgium) was used for image acquisition with the following settings: tube voltage 40 kV; tube current 250 μA; no additional filtration; isotropic voxel size 1.6 μm; number of projections 1800; averaging five frames/projection; and exposure time 1815 ms. The duration of the 360° acquisition for one sample was approximately 5.5 h. The average cartilage thickness was 2.4 mm [95%CI = (1.9; 3.0)]. Image reconstruction was performed with NRecon software (v1.6.10.4, Bruker microCT). Beam-hardening and ring-artifact corrections were applied during the reconstruction phase.

### Chondron selection and segmentation algorithm

Volumes of interest (VOIs) with sizes of 300 × 300 × Z (480 × 480 × Z_h_ μm^3^, Z_h_ being the height of the sample) were chosen for analysis, as middle of the image stacks as possible, but avoiding the imaging artifacts. A custom-made algorithm developed in Matlab (v.8.5, Mathworks, Natick, MA, USA) was applied to automatically select and segment the chondrons (see Supplementary material). Briefly, chondron selection was performed by assessing the amount of the volumetrically connected (within the 3D vicinity) voxels as possible chondron. A sub-VOI was then generated for each potential chondron to be volumetrically segmented. For each sub-VOI, 3D histogram equalization was applied to enhance the contrast between the chondron and the ECM. Then, a multiscale 3D local binary patterns–based method, adapted from^31^, ^32^, was used to segment the chondron itself. This method assesses the volumetric continuity of the voxels that are considered part of the identified chondron by fitting multiple spheres with different radii and evaluating the gray-level distribution calculated at their surface. As a preliminary criterion, a minimum threshold of segmented volume (400 μm^3^) was used to remove potential segmented artifacts.

### Manual verification of the segmentation

A second custom-made Matlab algorithm was used to manually verify the automatic segmentations. It contains a graphical user interface that visualizes the superposition of the original image and the segmented mask in a 3D orthogonal view from the center of the segmented volume (see Fig. S4 in the Supplementary material) allowing the user to determine the accuracy of the segmentations. The algorithm also enables the manual annotation and separation between segmented chondrons containing either a single cell or multiple cells (cluster).

### Algorithm validation

The intra-repeatability of the manual verification of the chondron selection was evaluated from 1000 segmentations from one sample (at two time points). To validate the performance of the automatic 3D segmentation script, 20 chondrons containing a single cell and 20 chondrons containing a cluster were randomly selected from different AC depths (range: 2–93% from the AC surface) from the manually verified automatic segmentations (22 chondrons from the healthy group, and 18 chondrons from the OA group). They were then segmented manually with MIMICS software (v.17.0.0.435, Materialise NV, Leuven, Belgium) by two independent users; the similarity of the manual and automatic segmentations was then evaluated by calculating a Dice similarity coefficient (DSC) for each chondron:

(1) $$DSC=\frac{2*A}{\left(B+C\right)}$$

where *A* is the sum of the common segmented voxels between the two compared segmentations, *B* is the sum of the voxels from the first segmentation, and *C* the sum of the voxels from the second segmentation. Finally, the average DSC [standard deviation (SD)] was calculated between the compared segmentations. Furthermore, the linear relationships between the automatic and manual segmentations were calculated (Fig. S5). The repeatability of the developed methodology and the manual segmentations are reported in the Supplementary material.

### Volumetric parameters

Chondron density was calculated from the number of chondrons selected by the script. For each sample, script-identified chondrons were checked to discover any false detection or eventual duplicates. Of all the segmentations, 500 chondrons/sample were randomly picked throughout the AC thickness for further investigation. After manual approval of the segmentation accuracy, the volume and sphericity^33^ of the accurately segmented chondrons were calculated as well as their depth (%) from the AC surface. These parameters were calculated using CTAn software's (v.1.15.4.0, Bruker microCT) individual object analysis. The sphericity, first introduced by Wadell^33^, was calculated as follows:

(2) $$Sph=\frac{\sqrt[{3}]{\pi }{\left(6V\right)}^{\frac{2}{3}}}{S}$$

where *V* is the object volume and *S* its surface area. A sphericity value of 1 refers to a sphere, and values smaller than 1 refer to non-spherical and complex 3D objects.

The depth from the AC surface was calculated as the distance between the AC surface and the centroid z_c_ coordinate of each chondron obtained with CTAn. The parameters were then divided into three AC depth zones (zone 1: 0–10%; zone 2: 10–40%; zone 3: 40–100% from the AC surface, similarly to previous literature^34^, ^35^) and grouped by the OARSI histological grades (OARSI grades 0–1.0, *n* = 6; OARSI grades 3.0–3.5, *n* = 6).

### Statistical analyses

Linear mixed models (SPSS, v.22.0, IBM SPSS, Armonk, NY, USA) were used to compare chondron density, volume, and sphericity between the different OARSI grade groups, separately in the three different zones. Chondron density, volume, and sphericity were treated separately as dependent variables, patient number was set as a subject, and OARSI grade group as a fixed variable. Furthermore, in volume and sphericity analyses, different models were used for all well-segmented chondrons, chondrons containing single cells, and chondrons containing clusters. A *P*-value smaller than 0.05 was considered statistically significant. The data in the figures are presented as means, with 95% CIs. The raw data {means (SD) and medians [interquartile range (IQR)]}, together with sample-wise visualizations of the morphological results, are presented in the Supplementary material.

## Results

### HMDS-based μCT imaging

**Fig. 2:**
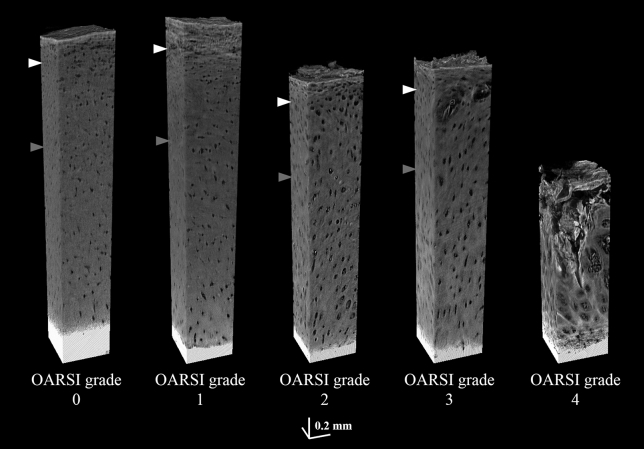
Volumetric visualizations of HMDS-dried osteochondral samples from OARSI grades 0–4 imaged with a desktop μCT. The white arrowhead refers to the border of zone 1 (0–10% from the AC surface) and zone 2 (10–40%); the gray arrowhead refers to the border of zones 2 and 3 (40–100%). The OARSI grade 4 sample shows erosion into the deep zone (i.e., the complete degeneration of the surface zone and advanced degeneration of the middle zone).

**Fig. 3:**
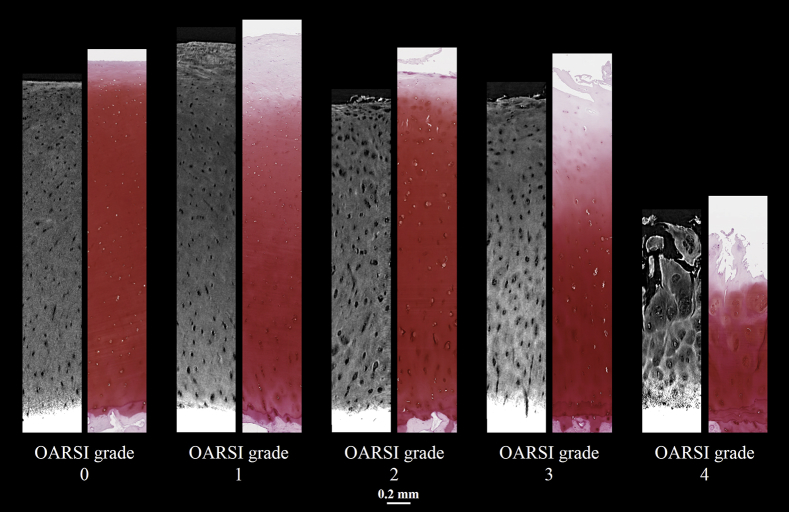
Example 2D images of HMDS-dried intact and osteoarthritic AC imaged with μCT, and the adjacent Safranin O–stained histological sections.

Volumetric visualizations of HMDS-dried osteochondral samples from OARSI grades 0–4 imaged with desktop μCT are shown in Fig. 2. When compared to conventional Safranin O–stained histological sections (Fig. 3), the features of OA in different OARSI grades are similarly visualized in both the 2D μCT slices and the histological sections. The cartilage thickness, however, appears slightly smaller in most of the μCT images than in the histological sections, and some inconsistencies in image quality occurred, primarily in the AC surface and at the cartilage-calcified cartilage interface.

### Automatic selection and segmentation performance

After the manual validation of the selected objects, a total of 12,804 chondrons were automatically segmented from all 12 samples. Out of 1000 segmented objects from one sample, only five selections were excluded during the first manual verification round, whereas during the second round, those same five were excluded along with three other selections, thus suggesting that the algorithm was able to accurately identify chondrons. Furthermore, 25% of the 500 chondrons/sample – the segmentation accuracy of which was determined using the manual verification algorithm – were approved for further analyses. The user input time for the verification of the 6000 chondrons (500 chondrons/sample) when using this new algorithm was roughly 48 h (on average 30 s per inspection), thus significantly decreasing the estimated time of ∼600 hours that a fully manual segmentation would require. The average DSC (SD) between the automatic and the two different manual segmentations were 0.85 (0.07) and 0.80 (0.07), respectively, and between the two manual segmentations, 0.78 (0.11).

### Chondron morphology vs OARSI grades

**Fig. 4:**
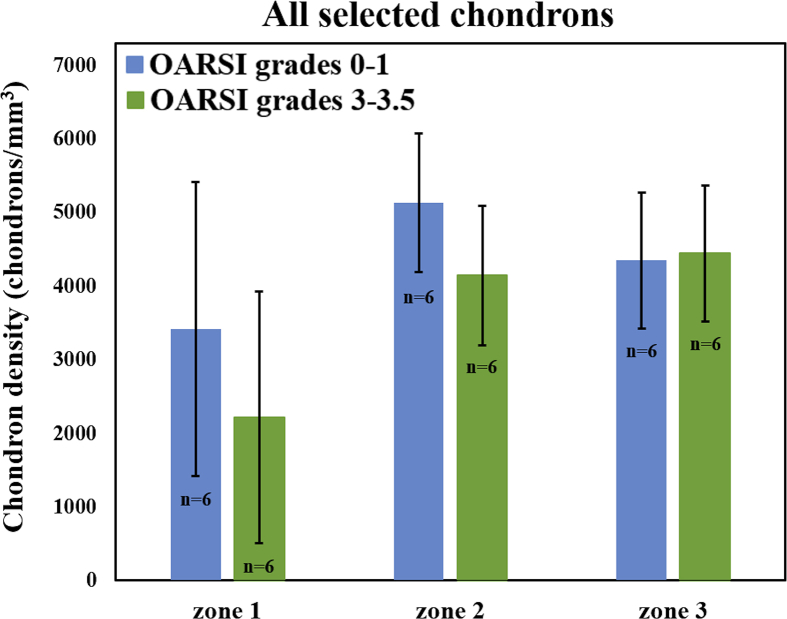
Average observed chondron densities with their 95% CIs in three AC depth zones (zone 1: 0–10%; zone 2: 10–40%; zone 3: 40–100% from the AC surface) grouped by the OARSI grades. No statistically significant difference was observed between the OARSI grade groups in any zone.

**Fig. 5:**
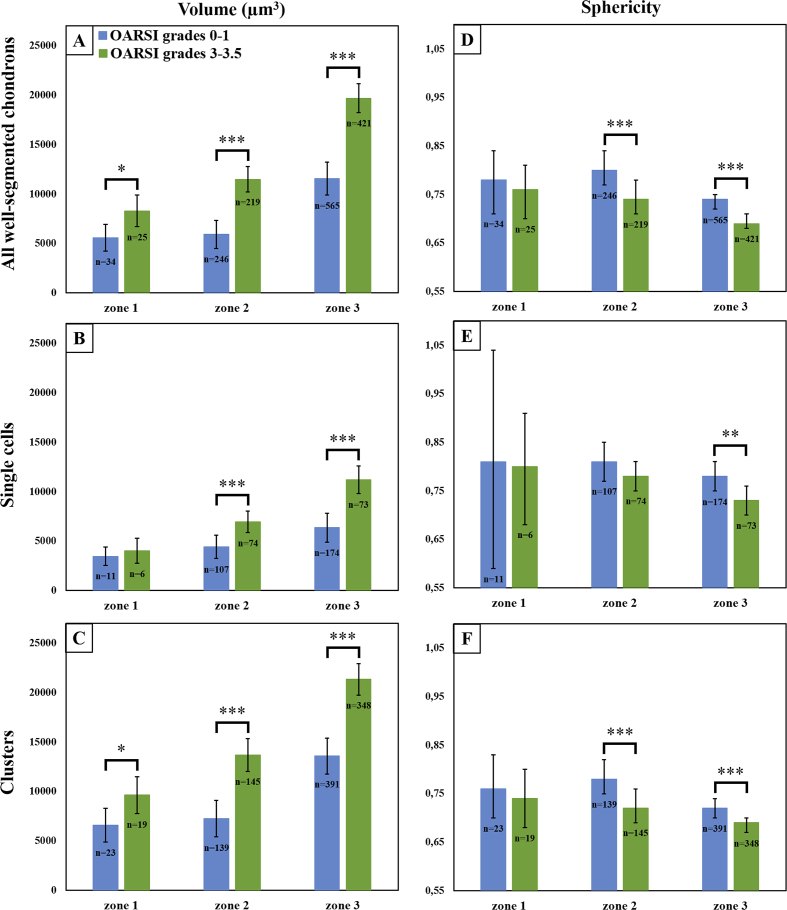
Morphological parameters of the well-segmented chondrons. The average chondron volumes (A–C) and sphericity values (D–F) with their 95% CIs in three AC depth zones (zone 1: 0–10%; zone 2: 10–40%; zone 3: 40–100% from the AC surface) grouped by the OARSI grades. A and D: all the well-segmented chondrons; B and E: chondrons containing only single cells; C and F: chondrons containing only clusters. *P < 0.05, **P < 0.01, ***P < 0.001.

**Fig. 6:**
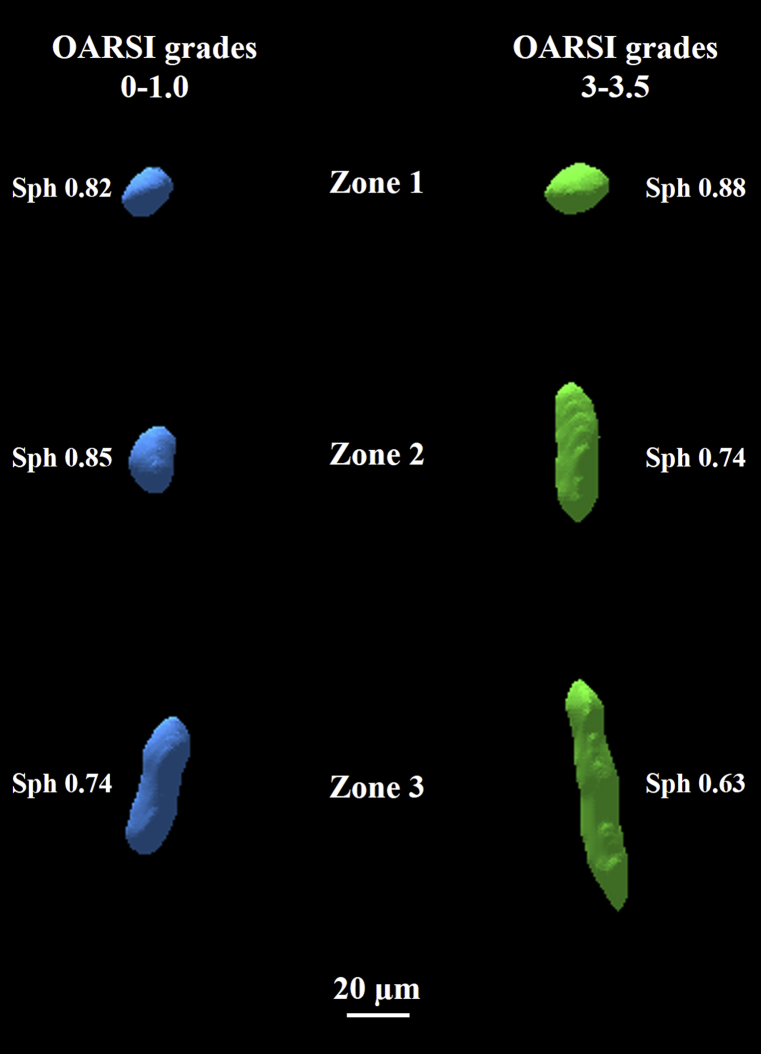
3D visualizations of representative segmented chondrons from both OARSI grade groups at different zones. Left: OARSI grades 0–1; right: OARSI grades 3–3.5.

The differences in chondron density between the two OARSI grade groups (Table I, Fig. 4) were not statistically significant in any depth zone. In contrast, the chondron volumes were significantly larger (zone 1: *P* < 0.05; zone 2: *P* < 0.001; zone 3: *P* < 0.001) in the OARSI grade 3.0–3.5 group compared to the OARSI grade 0–1.0 group [Fig. 5(A)]. Similar results were observed when considering only the chondrons containing clusters, but the differences were significant only in zones 2 and 3 (*P* < 0.001) for chondrons containing only single cells [Table II, Fig. 5(B) and (C)]. The chondron sphericity in zones 2 and 3 was significantly smaller (*P* < 0.001) in the OARSI grade 3.0–3.5 group compared to the OARSI grade 0–1.0 group [Table II, Fig. 5(D)]. Similar results were obtained for chondrons containing only clusters [Fig. 5(F)]. For chondrons containing only single cells, the trend was similar, but the differences were significant only in zone 3 (zone 2: *P* = 0.066 and zone 3: *P* = 0.002) [Fig. 5(E)]. Volumetric models of the successfully segmented chondrons from both OARSI grade groups are shown in Fig. 6.

**Table I:** Mean chondron density of the studied OARSI grade groups, together with the difference between means and *P* values from the linear mixed models

Chondron density (chondrons/mm^3^)					
		OARSI grades 0–1	OARSI grades 3–3.5	Difference (95% CI)	*P*
		Mean (SE)∗	Mean (SE)∗		
All selected chondrons	Zone 1	3418 (756)	2219 (755)	1199 (−1224; 3621)	0.291
	Zone 2	5131 (425)	4143 (425)	988 (−351; 2326)	0.131
	Zone 3	4349 (416)	4444 (416)	−95 (−1405; 1215)	0.875

SE = standard error.

∗ Estimated means.

**Table II:** Mean volumes and sphericities of the studied OARSI grade groups, together with the differences between means and *P* values from the linear mixed models

		OARSI grades 0–1	OARSI grades 3–3.5	Difference (95% CI)	*P*
		Mean (SE)∗	Mean (SE)∗		
**Volume (μm**^**3**^**)**					
All well-segmented chondrons	Zone 1	5578 (681)	8286 (794)	2708 (613; 4803)	0.012
	Zone 2	5909 (611)	11,481 (563)	5572 (4287; 6857)	<0.001
	Zone 3	11,560 (650)	19,686 (822)	8126 (6625; 9626)	<0.001
Single cells	Zone 1	3465 (437)	4024 (592)	559 (1010; 2128)	0.459
	Zone 2	4410 (484)	6944 (490)	2535 (1294; 3775)	<0.001
	Zone 3	6354 (529)	11,202 (654)	4848 (3274; 6421)	<0.001
Clusters	Zone 1	6589 (841)	9632 (926)	3043 (514; 5571)	0.020
	Zone 2	7244 (740)	13,690 (661)	6446 (4815; 8077)	<0.001
	Zone 3	13,576 (746)	21,346 (689)	7770 (6043; 9497)	<0.001
**Sphericity**					
All well-segmented chondrons	Zone 1	0.78 (0.02)	0.76 (0.02)	0.02 (−0.05; 0.09)	0.471
	Zone 2	0.80 (0.02)	0.74 (0.01)	0.06 (0.04; 0.09)	<0.001
	Zone 3	0.74 (0.01)	0.69 (0.01)	0.05 (0.03; 0.06)	<0.001
Single cells	Zone 1	0.81 (0.04)	0.80 (0.04)	0.02 (−0.19; 0.22)	0.775
	Zone 2	0.81 (0.01)	0.78 (0.01)	0.03 (0.00; 0.06)	0.066
	Zone 3	0.78 (0.01)	0.73 (0.01)	0.05 (0.02; 0.08)	0.002
Clusters	Zone 1	0.76 (0.03)	0.74 (0.02)	0.02 (−0.05; 0.09)	0.469
	Zone 2	0.78 (0.02)	0.72 (0.02)	0.06 (0.03; 0.08)	<0.001
	Zone 3	0.72 (0.01)	0.69 (0.01)	0.03 (0.02; 0.05)	<0.001

∗ Estimated means.

## Discussion

This study has presented a novel HMDS-based sample processing protocol for osteochondral samples, which permits high-resolution imaging of chondrons at full AC thickness using conventional desktop μCT. We also developed a semi-automatic 3D selection and segmentation algorithm and applied this protocol to quantify the morphology of intact and osteoarthritic human AC chondrons in 3D. The proposed method is time-efficient and provides a high-throughput approach to investigate changes that occur in AC chondrons in OA with minimum user input.

Unlike previous methods for 3D chondrocyte/chondron imaging, such as confocal microscopy^1^, ^13^ and synchrotron-based μCT^15^, our protocol is relatively fast and easy to apply, and it does not require any extra equipment or specific stains. Our protocol also allows for imaging considerably larger volumes than e.g., with confocal microscopy. HMDS was selected as a drying solution because it allows the complete drying of the cartilage, thus enabling contrast in the μCT images to arise from the natural X-ray attenuation of the tissue itself, rather than from external contrast agent distribution.

The second objective of this study was to develop and validate an automated 3D selection and segmentation algorithm for chondrons imaged with μCT. The main advantage of this algorithm is that it allows rapid 3D analyses of multiple chondrons and, unlike threshold-based algorithms, it takes into account the volumetric information of the chondron. This approach is also easy to implement in other laboratories and for other segmentation purposes. When compared to manual segmentation, this new algorithm is roughly 12 times faster, the only user task being to check the performance of the segmentation provided by the script. From the 500 chondrons/sample selected for manual inspection, 25% were approved and further analyzed. This seemingly low percentage was primarily the result of the cartilage surface area, where the segmentation was fairly difficult – even manually – due to inconsistencies in image contrast and the chaotic-like cartilage surface morphology (especially in the case of OA samples). The area near the interface between the non-calcified and calcified cartilage is also challenging to segment because of beam-hardening which results in streaking artifacts and incorrect attenuation values near the interface. In this protocol, these artifacts occurred because of large differences in the X-ray attenuation between the non-calcified and calcified tissue, and could be prevented by decalcification of the sample. While the percentage of approved chondrons easily could have been increased by adding conditions related to locations in the algorithm, we decided not to adjust the script to avoid missing any potential chondrons in these challenging areas. The second reason for some of the inaccurate segmentations was due to brighter areas (the remnants of chondrocytes) inside or at the border of chondrons, which affected the automatic process. The accuracy of the automatic segmentations was visually evaluated from three orthogonal 2D views rather than from the full 3D volume. While the latter method would be more accurate, it would also be more time-consuming because of higher computational costs related to the generation and rotation of 3D models.

The average DSC calculations were used to evaluate the performance of the automatic 3D segmentation algorithm, as in a previous publication^36^. The average DSC (SD) between the automatic and manual segmentations for the first and second segmenters were 0.85 (0.07) and 0.80 (0.07), respectively, which suggests that both segmenters agreed well with the automatic segmentation; we can therefore conclude that the script for automatic segmentation was accurate. The average DSC between the two segmenters, however, was slightly lower [0.78 (0.11)], which may have indicated that the manual segmentation was not completely inter-reproducible.

The developed segmentation script was able to identify a total of 12,804 chondrons from all 12 samples. Even though the segmentation of chondron borders occasionally failed, the script was able to automatically identify a large number of chondrons. The chondron density in zones 1 and 2 was observed to be slightly lower in the OARSI grade 3–3.5 group compared to the OARSI grade 0–1 group, but not at a statistically significant level. In zone 3, chondron density was similar between the two OARSI grade groups. The absolute chondron density results in the middle and deep AC were in the same range as previously reported^11^, ^37^, ^38^, although our density values in zone 1 were considerably lower than what previous studies have found. As mentioned above, zone 1 was problematic for the segmentation script, which could explain the high variation and seemingly low density values in that zone. Previous literature shows that in healthy AC, chondrocyte density seems to decrease at greater tissue depths^11^, ^37^, ^38^, which we also observed in our results when not considering zone 1.

From the morphological analysis, we found that the chondrons were significantly larger in the OARSI grade 3–3.5 group. These results concur with previous studies that have reported increasing chondron volumes with OA^8^, ^9^. The observed hypertrophic morphological changes in chondrons could be explained by their upregulated metabolism induced by OA activity^7^, ^39^. Horikawa *et al.* (2004) suggested another explanation for chondron hypertrophy; the authors showed that the volume ratio of the pericellular microenvironment to chondrocyte increases with OA^40^. We observed in our study that the chondrons were less spherical in the OARSI grade 3–3.5 group in zones 2 and 3, which suggests that the increase in chondron size did not occur evenly in all directions, but the hypertrophic changes made the chondrons more cylindrical. Alexopoulos *et al.* (2005) have previously shown that the Young's modulus of PCM is significantly lower than that of the surrounding ECM^41^. This finding indicates that the PCM shape might be modulated by the properties of the surrounding tissue and could partly explain the morphological changes in the chondrons observed in our study.

In zone 1, statistically significant differences in chondron volume and sphericity were not systematically observed, which indicates chondron hypertrophy to be less common in the superficial AC layer, although this observation could also be explained by either a deficient segmentation of smaller chondrons or as a sample processing artifact in the superficial AC layer. The superficial layer remains a challenge for chondron segmentation, mainly due to the shrinkage of the cartilage surface during the HMDS drying protocol. Poole *et al.* (1987) have shown that surface chondrons do not have the pericellular capsule that surrounds the pericellular matrix of the chondrons in the middle and deep AC^6^. This could explain why surface chondrons are more prone to shrinkage during the drying. Another previous study has also shown that in OA, type VI collagen expression and distribution is increased in the lower-middle and upper-deep zones but not in the upper zones^42^. This finding suggests that the protective barrier of the chondrons in the middle and deep AC is stronger than in surface chondrons, thus making the surface chondrons more vulnerable to changes caused by the sample processing.

Previously reported chondron/chondrocyte/PCM volumes have shown relatively large variation^1^, ^11^, ^13^, ^15^, ^37^, ^38^, ^40^, ^43^, most likely due to the different species, sample processing, and imaging modalities used. In a comparison of our volumetric results of chondrons containing single cells to those of previous human studies^1^, ^11^, ^37^, ^38^, ^40^, our results were more or less in the same range, although our observed values were slightly larger. The lack of studies that have quantitatively investigated the morphology of clusters prevents a thorough comparison of our observations with the previous studies. Finally, it is important to note that chondrons are dynamic^6^ (i.e., they respond to changes in their environment), and therefore their morphological properties may differ depending on the OA stage; a comparison of the results obtained from different modalities thus may not be directly relevant.

One major limitation of this study was the unfortunate freeze/thaw cycles before the sample processing. This could not be prevented because of the distant origin of the samples (Montreal, Canada) and because they had been used in other studies as well. All the samples did go through the same processes, however, and therefore the results obtained in this study should be comparable to each other. Although HMDS has relatively low surface tension and the ability to cross-link proteins^26^, some shrinking was observed in the cartilage because of the drying, with zone 1 being affected the most. The sample processing protocol could thus be developed further to minimize (and potentially even prevent) the cartilage shrinking. With the segmentation verification system used in this study, the differentiation between the chondrons containing a single cell and those with a cluster was not completely accurate, as some clusters may not have been visible in the three orthogonal 2D views, and thus they could have been labeled as chondrons containing a single cell. To avoid any misinterpretation in the verification process arising from the current system, verification of the automatic segmentations could be conducted from the full 3D volume instead of from the orthogonal views. Another limitation was that our sample size was relatively small, which was partly due to the novel method we were testing. Thus, a larger number of samples (and more thorough set of OARSI grades) should be included in the future studies. Because the selection of cores was based on their histological grades, some variation in core locations occurred. Separate datasets were not used in the validation of the methodology and the chondron morphometry assessment, which could also be considered a minor weakness. Finally, the zone-division approach we used is accurate only for samples with remaining cartilage surface (OARSI grades 0–3.5). Other zone-division methods (e.g., those based on polarized light microscopy) would be necessary if investigating more advanced OA grades.

To conclude, we have developed a novel sample processing protocol for imaging AC chondrons in 3D with a conventional desktop μCT, and a validated semi-automatic algorithm for chondron selection and segmentation for morphological 3D chondron analysis. The protocol and algorithm were applied to quantify the morphology of human AC chondrons in 3D and to assess the differences between intact and OA samples. We did not find a statistical difference in chondron density between the intact and OA samples but chondrons did become larger and more elongated in OA, which indicates that the catabolic events in the surrounding ECM had an effect on chondron morphology. The HMDS-based sample processing protocol also has the potential for more than chondron imaging: it has been suggested that this method could be useful for 3D quantification of the collagen distribution in AC as well^44^. This new μCT protocol provides a new approach to spatially evaluate not only chondron/chondrocyte properties but also ECM macromolecule distribution in 3D during different phases of OA.

## Author contributions

Conception and design: IK, JT, MAF, SSK, IH, MG, EQ, LR, KPP, MDB, HJN, SS.

Analysis and interpretation of the data: IK, JT, MAF, SSK, MH, LR, HJN, SS.

Drafting of the article: IK, JT.

Critical revision of the article for important intellectual content: IK, JT, MAF, SSK, IH, SK, MG, EQ, MH, LR, KPP, MDB, HJN, SS.

Final approval of the article: IK, JT, MAF, SSK, IH, SK, MG, EQ, MH, LR, KPP, MDB, HJN, SS.

Provision of study materials or patients: IH, MG, EQ, MDB.

Collection and assembly of data: IK, IH, SK.

## Conflict of interest

• IH has received Ph.D student award for International Internship in Finland from MÉDITIS training program, Canadian Arthritis Society.
• MG and EQ are employed by Biomomentum.
• HJN has received Academy of Finland grant, has several patent publications (Univ. of Oulu, Univ. of Helsinki, Philips Healthcare, Photono Oy, SWAN Cytologics, Revenio), and also receives royalties from them.
• SS has received grants from Academy of Finland, European Research Council, and Sigrid Juselius Foundation, and has one pending patent application.
• Other authors (IK, JT, MAF, SSK, SK, MH, LR, KPP, and MDB) report no conflicts of interest.

## Declaration of funding

The financial support from the Academy of Finland (grants no. 268378, 273571, 311586); Sigrid Juselius Foundation; European Research Council under the European Union's Seventh Framework Programme (FP/2007-2013)/ERC Grant Agreement no. 336267; and the strategic funding of the University of Oulu are acknowledged.

## Role of the funding source

Funding sources are not associated with the scientific contents of the study.
