# Supplementary material for: In vitro method for 3D morphometry of human articular cartilage chondrons based on micro-computed tomography
Source: Osteoarthritis Cartilage. 2018 Aug;26(8):1118–26. doi: 10.1016/j.joca.2018.05.012 (PMC6058088; doi:10.1016/j.joca.2018.05.012)
Supplement: Multimedia component 1 [file mmc1.docx]

Kestilä I *et al.* Articular Cartilage Chondrons in µCT

**Supplementary material**

**The repeatability analyses**

*The reproducibility of manual segmentation*


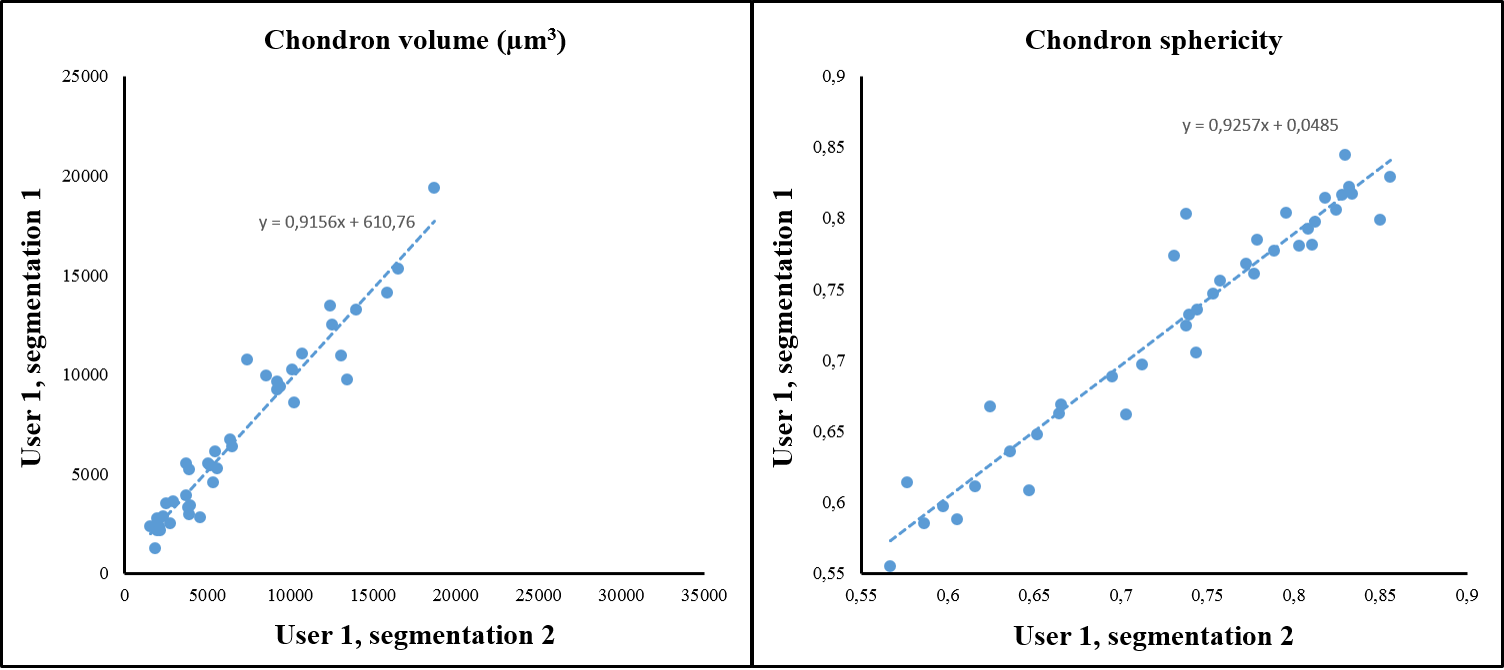


**Figure S1.** The reproducibility of the manual segmentation visualized as linear relationship between the segmentations performed at two different time points. 40 chondrons were selected: 22 chondrons from the healthy group (11 single cells and 11 clusters) and 18 chondrons from the OA group (9 single cells and 9 clusters). Left: Volume; Right: Sphericity. The DSC (SD) between the first and the second manual segmentations is 0.91 (0.06).

*The repeatability of the chondron morphology analyses*


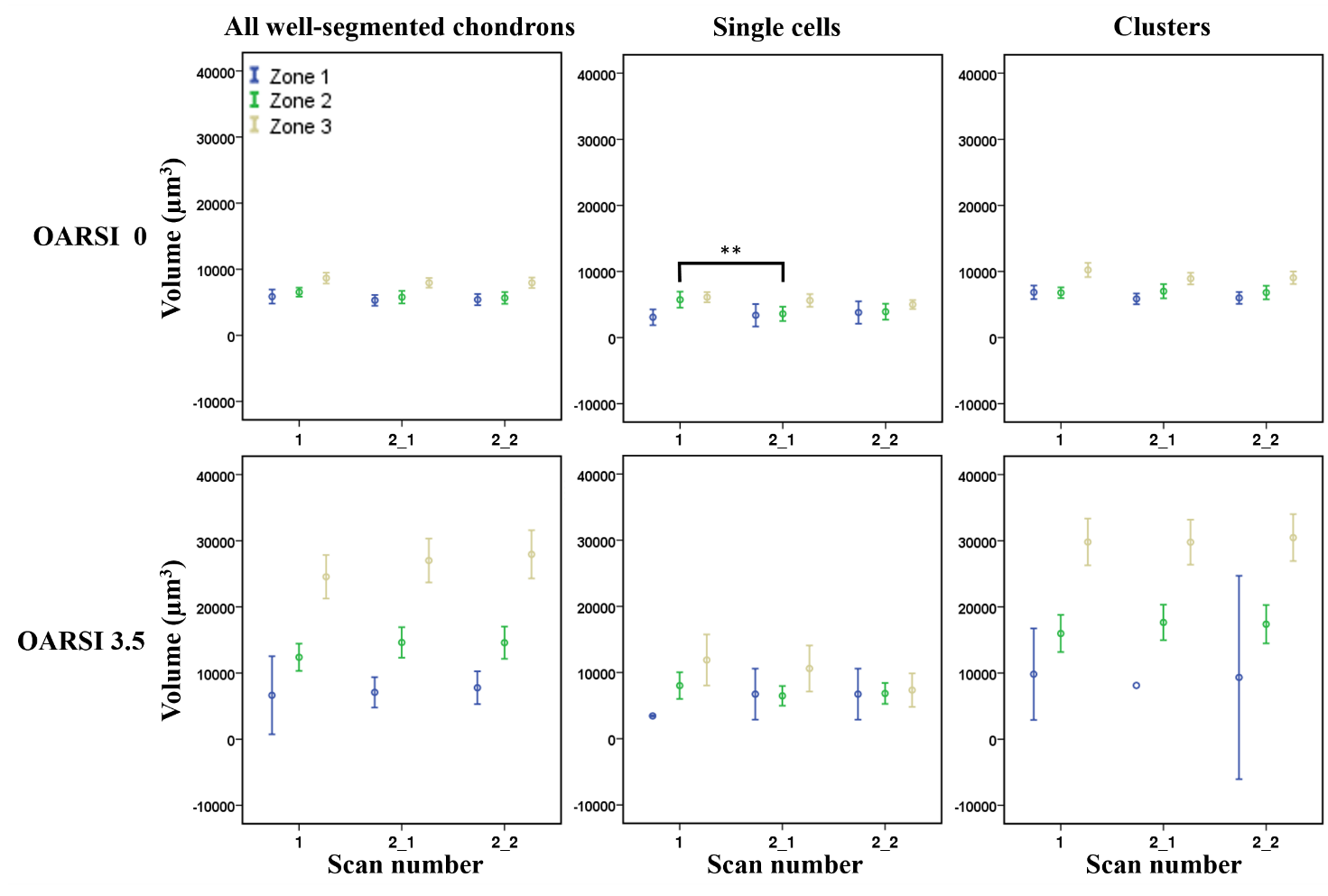


**Figure S2.** The average chondron volumes of the repeated scans with their 95% confidence intervals. All the well segmented chondrons on the left, single cells in the middle, and clusters on the right. Top row: the repeated analyses of the OARSI grade 0 sample; Bottom row: the repeated analyses of the OARSI grade 3.5 sample. The scan number 1 refers to the first scan, scan number 2_1 to the first analyses of the second scan, and 2_2 to the second analyses of the second scan. The exact *p*-values are shown in Table S1.

**Table S1.** The *p*-values of the differences in volume (µm^3^) from the independent samples t-test. Statistical comparisons were conducted between the first scan and the first analyses of the second scan (1 vs 2_1), and between the first and the second analyses of the second scan (2_1 vs 2_2).


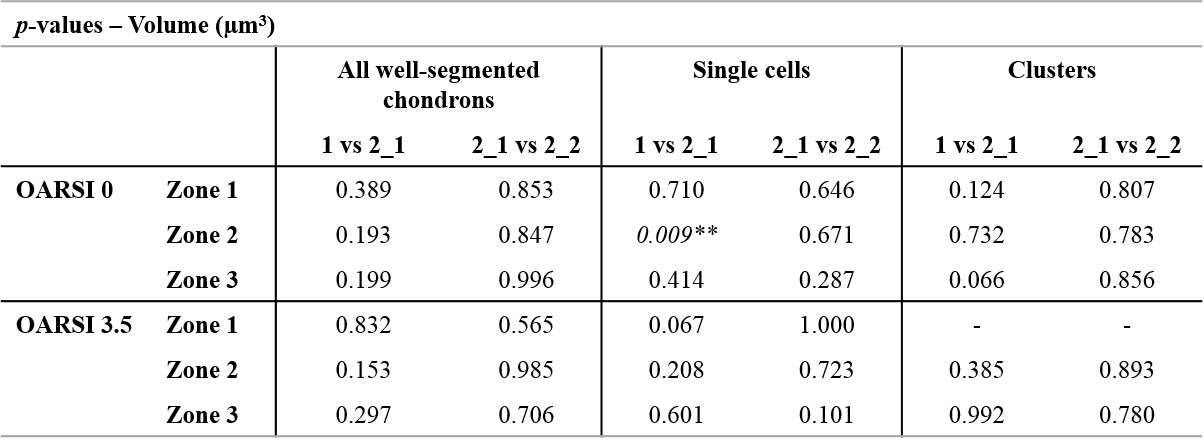


**
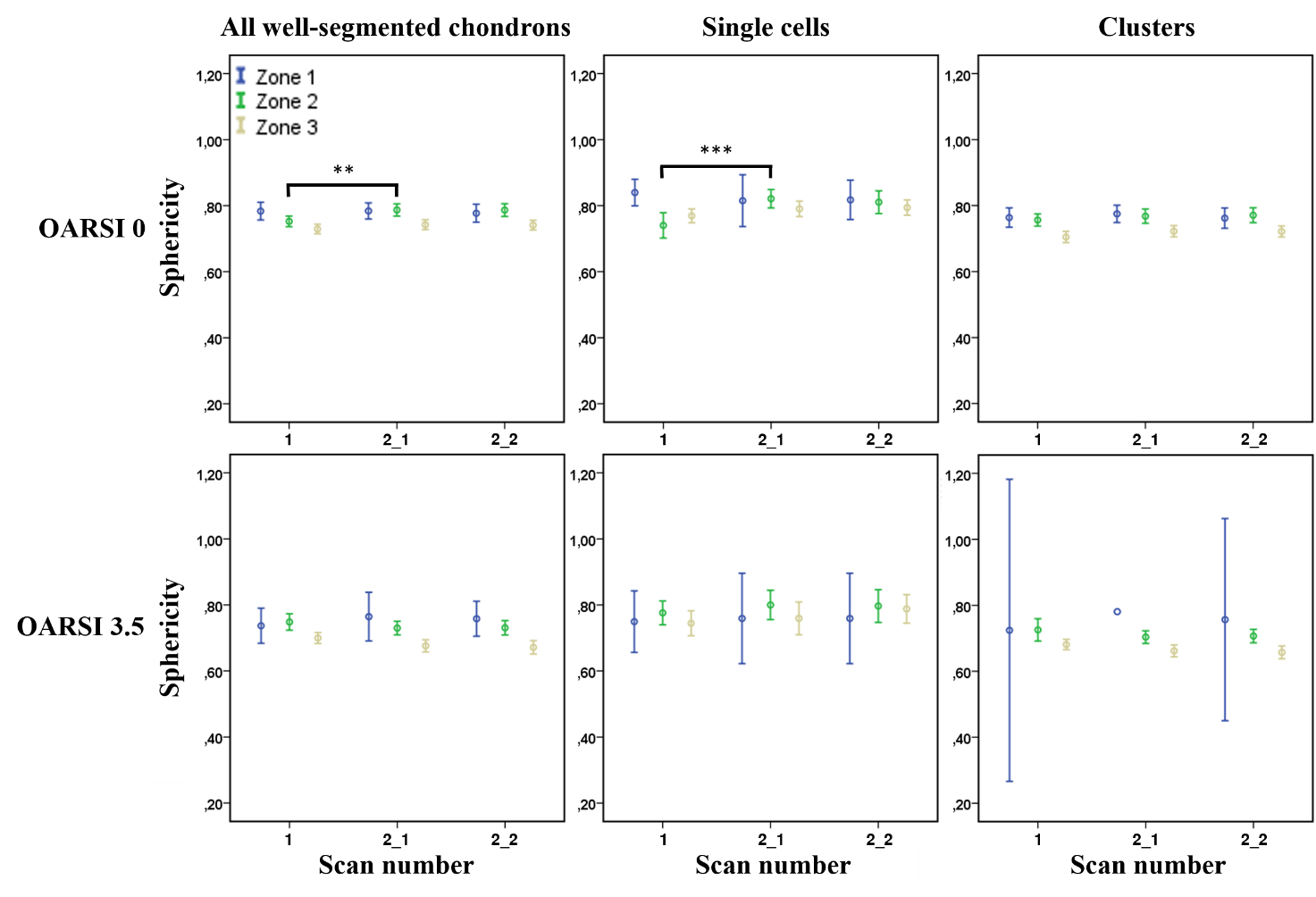
**

**Figure S3.** The average chondron sphericities of the repeated scans with their 95% confidence intervals. All the well segmented chondrons on the left, single cells in the middle, and clusters on the right. Top row: the repeated analyses of the OARSI grade 0 sample; Bottom row: the repeated analyses of the OARSI grade 3.5 sample. The scan number 1 refers to the first scan, scan number 2_1 to the first analyses of the second scan, and 2_2 to the second analyses of the second scan. The exact *p*-values are shown in Table S2.

**Table S2.** The *p*-values of the differences in sphericity from the independent samples t-test. Statistical comparisons were conducted between the first scan and the first analyses of the second scan (1 vs 2_1), and between the first and the second analyses of the second scan (2_1 vs 2_2).


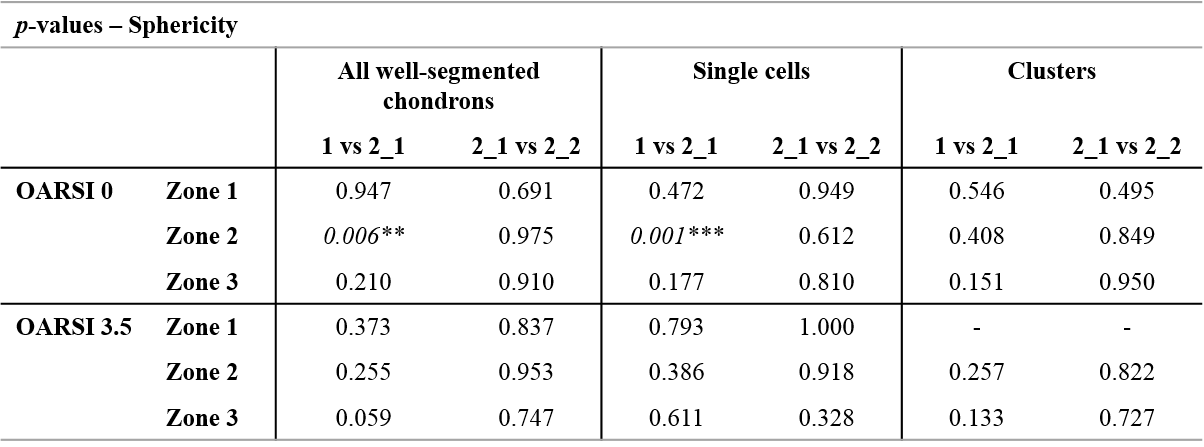


**Raw data**

**Table S3.** Raw data tables – Chondron density. The means (SD) and medians (IQR) are presented in both of the OARSI grade groups in all of the three depth zones.


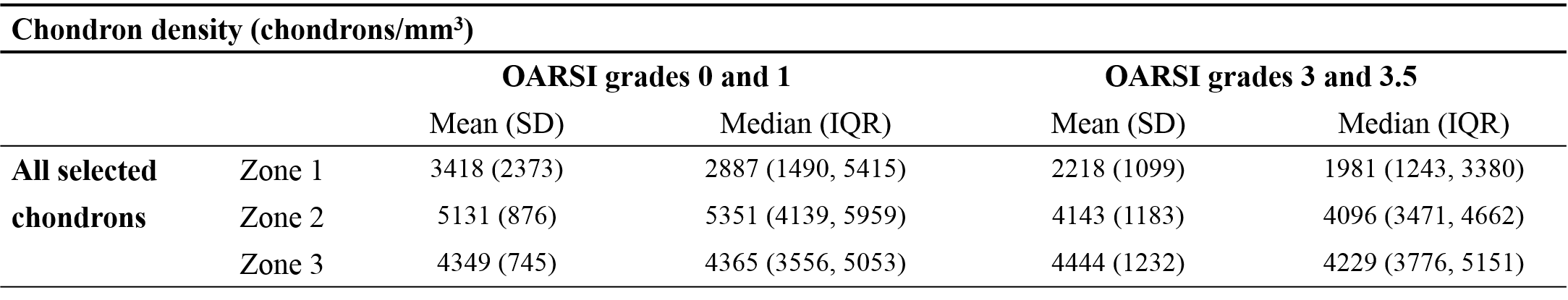


**Table S4.** Raw data tables – Volume (µm^3^). The means (SD) and medians (IQR) are presented in both of the OARSI grade groups in all of the three depth zones.


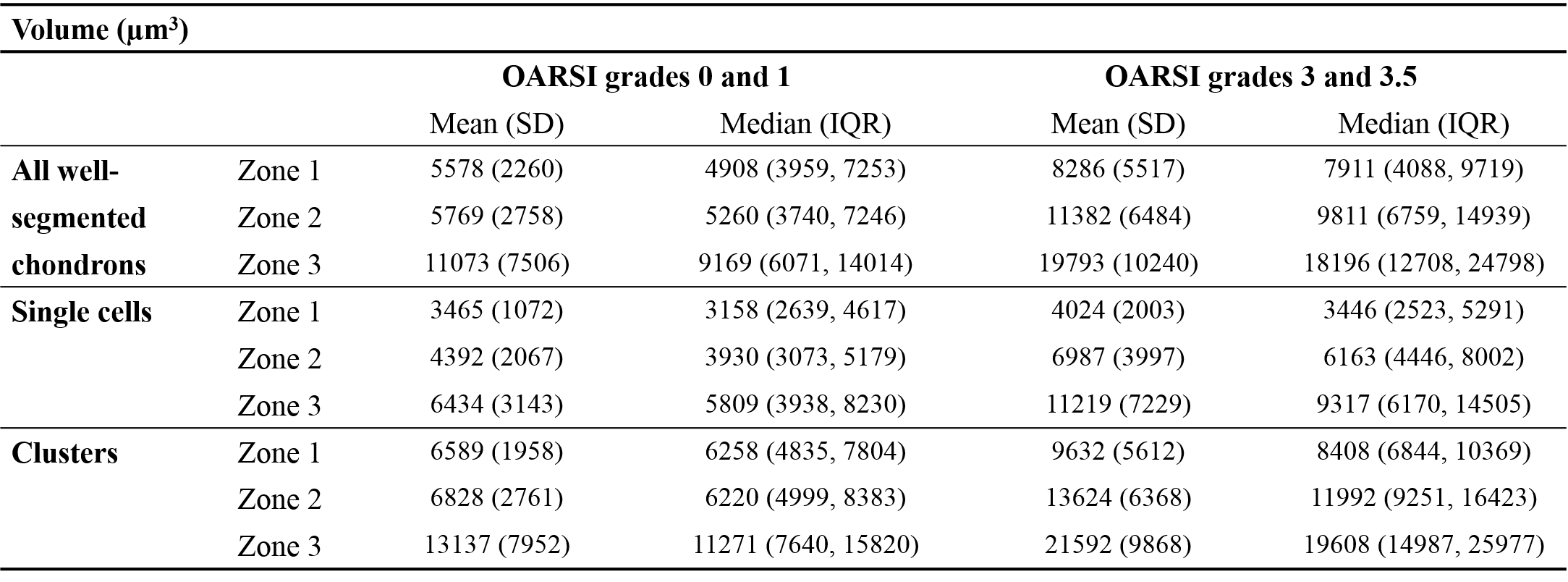


**Table S5.** Raw data tables – Sphericity. The means (SD) and medians (IQR) are presented in both of the OARSI grade groups in all of the three depth zones.


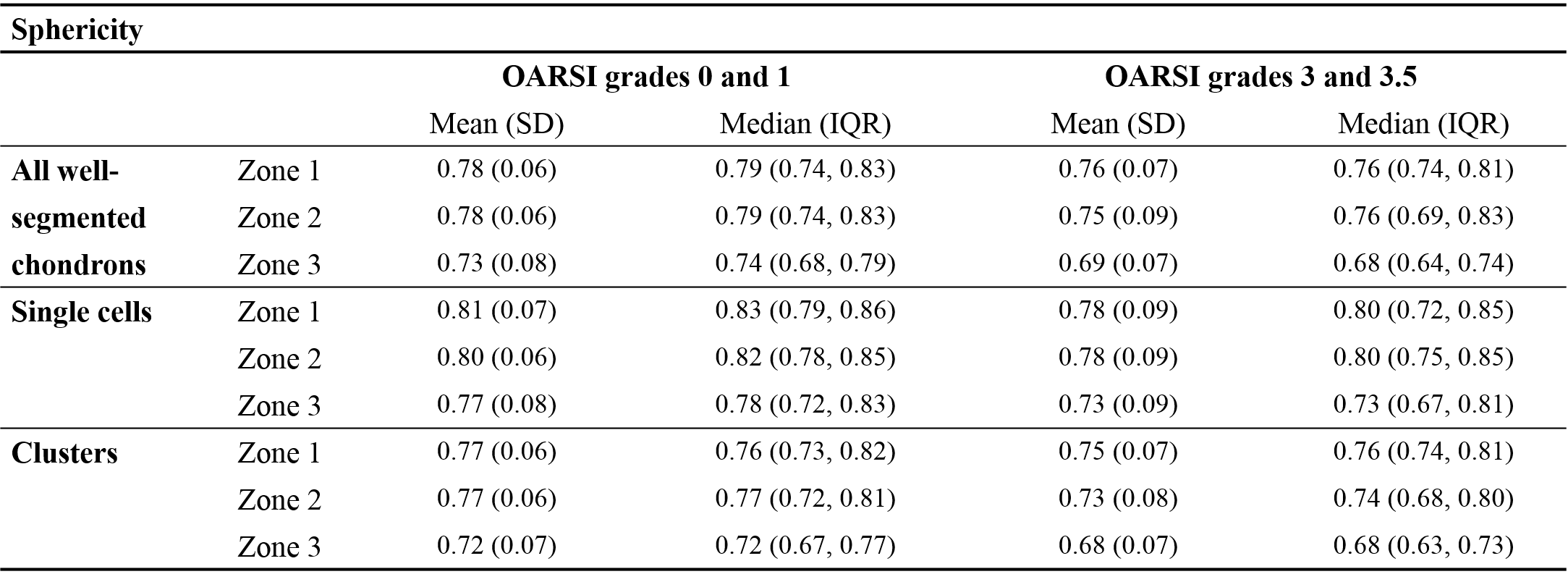


**Chondron selection and segmentation algorithm**

**Algorithm 1.** Finding the chondrons within one scanned sample. An iteration can be implemented in the algorithm by having an increasing δ (Step 1), considering more voxels as potential lacunae.

Input Stack of images I obtained by µCT

Output List C of voxel coordinates within potential chondrons (one seed per chondron)

Step 1 Filter out the non-lacuna space by Otsu method

- 1. L = ɸ

1.2 Compute global threshold by Otsu method G_otsu_

1.3 $\forall$δ ∈ {0.50… 0.95}

If I < (δ * G_otsu_)

L = 1

End

Step 2 Finding the potential chondrons within the mask L

2.1 C = ɸ

2.2 For p(x,y,z) ∈ L

Let K_l_ ∈ ℕ, defining the size of a moving cubic window (*e.g* K_l_ = 3, size 7x7x7) and A the minimum percentage of voxels within this window considered as lacuna (*e.g* A=80%)

If $(\sum_{x-K_{l}}^{x+K_{l}} \sum_{y-K_{l}}^{y+K_{l}} \sum_{z+K_{l}}^{z+K_{l}} p)$ *≥ A*(2K_l_+1)^3^*

L _=_ L - p(x-K_l_:x+K_l_,y-K_l_:y+K_l_,z-K_l_:z+K_l_)

C = C + {p(x,y,z)}

End

End

**Algorithm 2.** Segmentation of a potential chondron. Adapted volumetrically from^1^

Input Stack of images I obtained by µCT, list C of voxel coordinates within potential chondrons

Output Segmented chondrons

Step 1 Generating a volume of interest for the considered potential chondron

1.1 $\forall$c(x,y,z) ∈ C, a seed of potential chondron

let J a cubic subspace ∈ I, Kch ∈ ℕ defining the size (*e.g* Kch = 50, size 101x101x101) in order to fit the whole potential chondron within it

J = I (x-Kch:x+Kch,y-Kch:y+Kch,z-Kch:z+Kch)

1.2 J_eq_ = histogram equalization (J)

Step 2 Assessing the connectivity of the lacuna

2.1 S = S1 = S2 = ɸ

Let G_k,1_ be any of the k-th neighbor at radius 1 from a center voxel {G_v_}; G_m,2_ be m-th neighbor at radius 2 from G_v_. k=0,1,…25 and m=0,1,…77

2.2 Compute threshold value in J_eq_ by Otsu method G_otsu_

2.3 For Gv ∈ J_eq_

If ∃ Gv ≤ 0.95* G_otsu_ and G_k,1_ ≤ 0.95* G_otsu_

S1 = S1 + {Gv}

End

End

2.4 Let R be the max distance used for connectivity test

For Gv ∈ J_eq_

If ∃ (Gv > 0.95* G_otsu_) and (G_k,1_ ≤ 0.95* G_otsu_) and (G_m,2_ ≤ 0.95* G_otsu_),

and $\left\| G_{k,1}-G_{m,2} \right\|\leq R$

S2 = S2 + {Gv}

End

End

2.5 S = S1 + S2

Step 3 Post-processing of the segmentation

3.1 S3 = ɸ

Let T_n_ be the percentage threshold for eventual cell remnants within the potential chondron (*e.g* Tn = 85%)

3.2 If J_eq_ ≥ (T_n_ * max(J_eq_))

S3 = 1

End

3.3 S = S + S3

3.4 S = volumetric_median_filtering (S), using a filtering cubic radius Rc allowing to connect potential cell remnants to the lacuna (e.g Rc=5)

3.5 S = volumetric_fill (S), function filling holes within a segmented volumetric object

3.6 S = keep_center_object (S), function removing all objects ∈ S not connected to the original seed c(x,y,z)

3.6 S = volumetric_remove_tails (S), function removing abnormally “flat” subvolumes

3.7 S = volumetric_dilatation (S)

Let Vt being the minimum volume to consider the segmentation plausible

3.8 If $\sum S \geq Vt$

S = S, the segmentation is considered for further analysis

Else

S = ɸ, the segmentation is not considered any further

End

**Manual chondron validation interface**


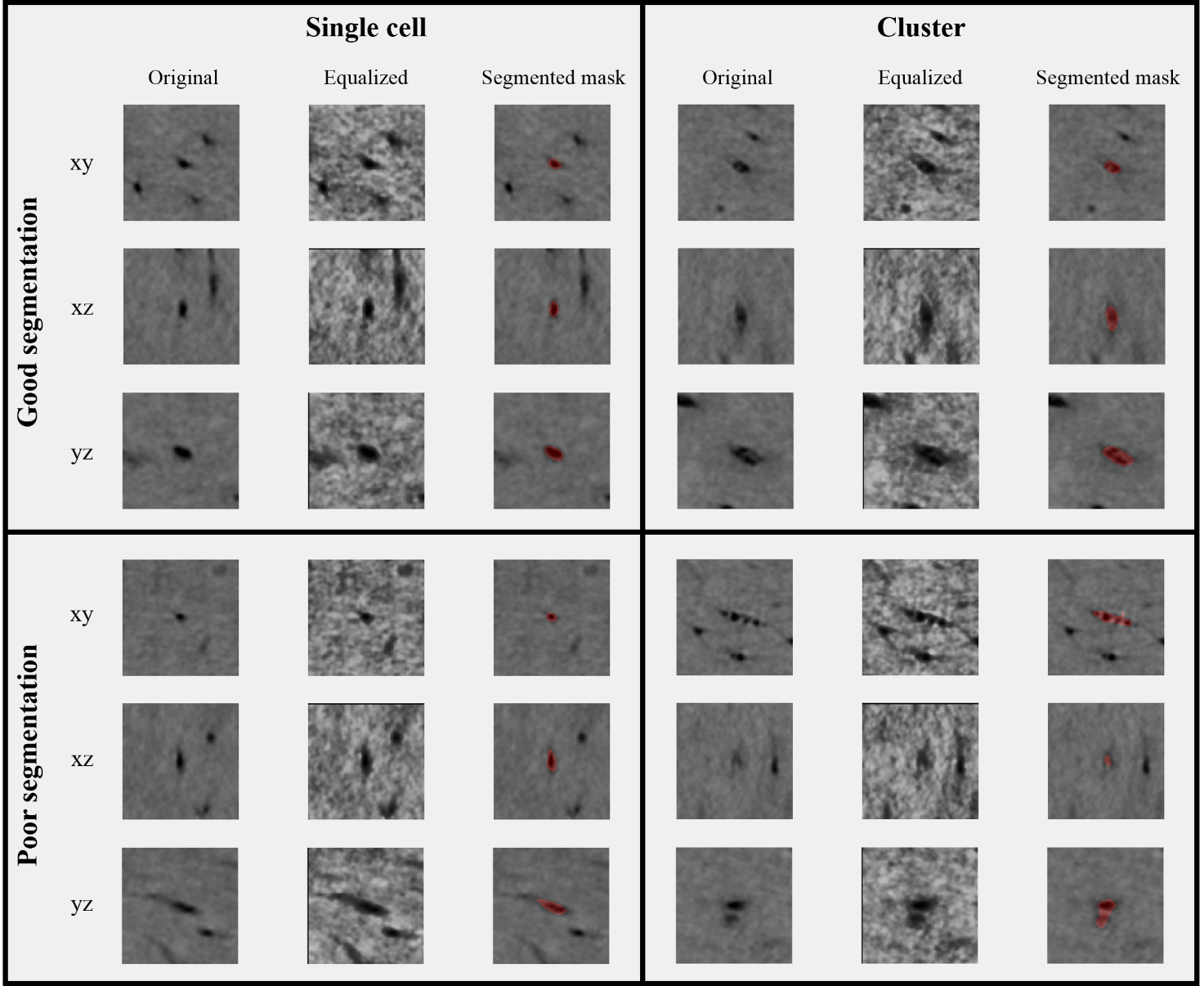


**Figure S4.** The example visualizations of the graphical user interfaces used in the manual verification of the automatic segmentations. On the left, examples of well segmented (top) and poorly segmented (bottom) single cells, and on the right, examples of well segmented (top) and poorly segmented (bottom) clusters.

**The performance of the automatic segmentation**

**
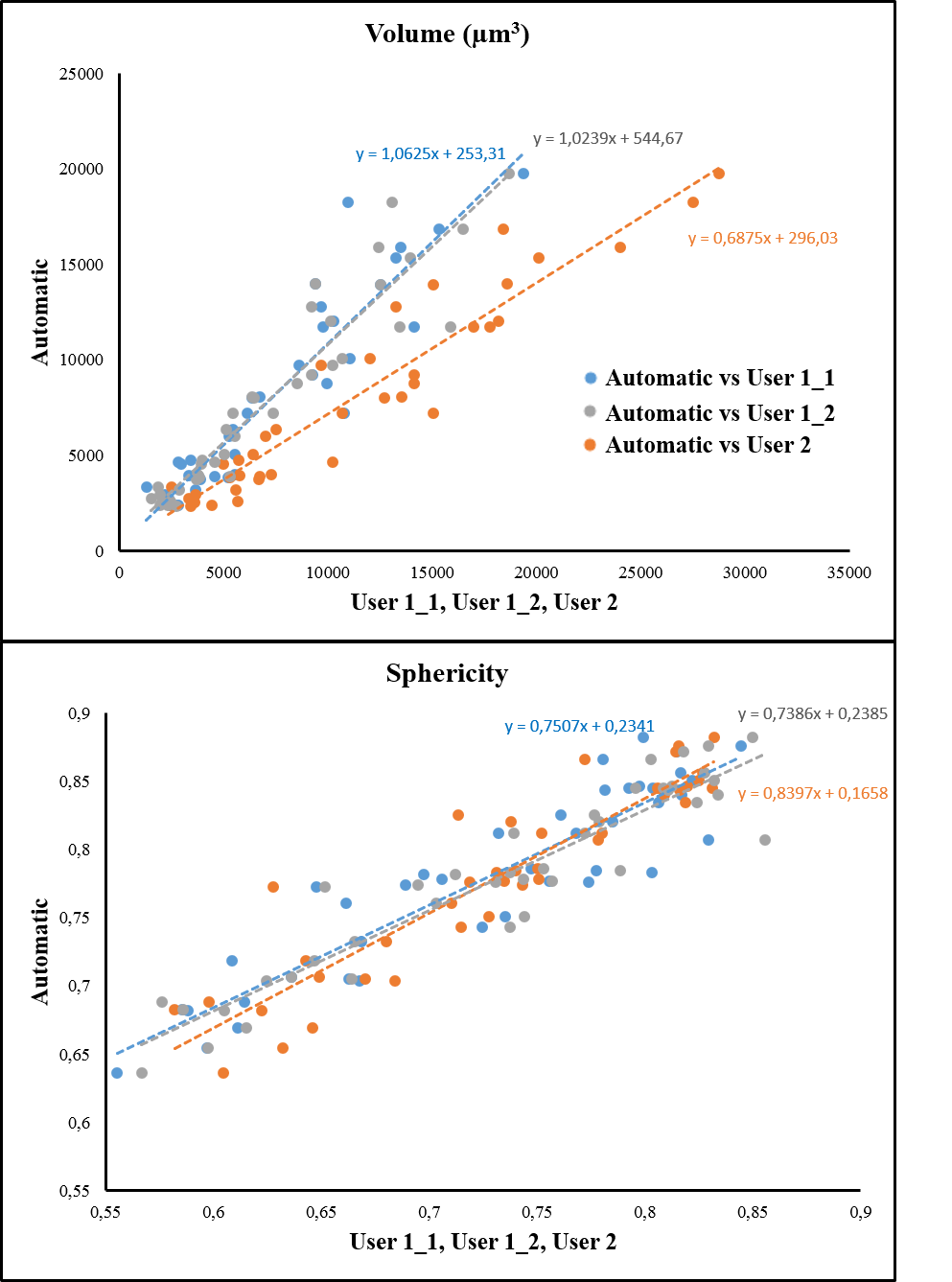
**

**Figure S5.** The linear relationships of the volume (top) and sphericity (bottom) results between the automatic and manual segmentations.

**Sample-wise visualizations of the morphological results**


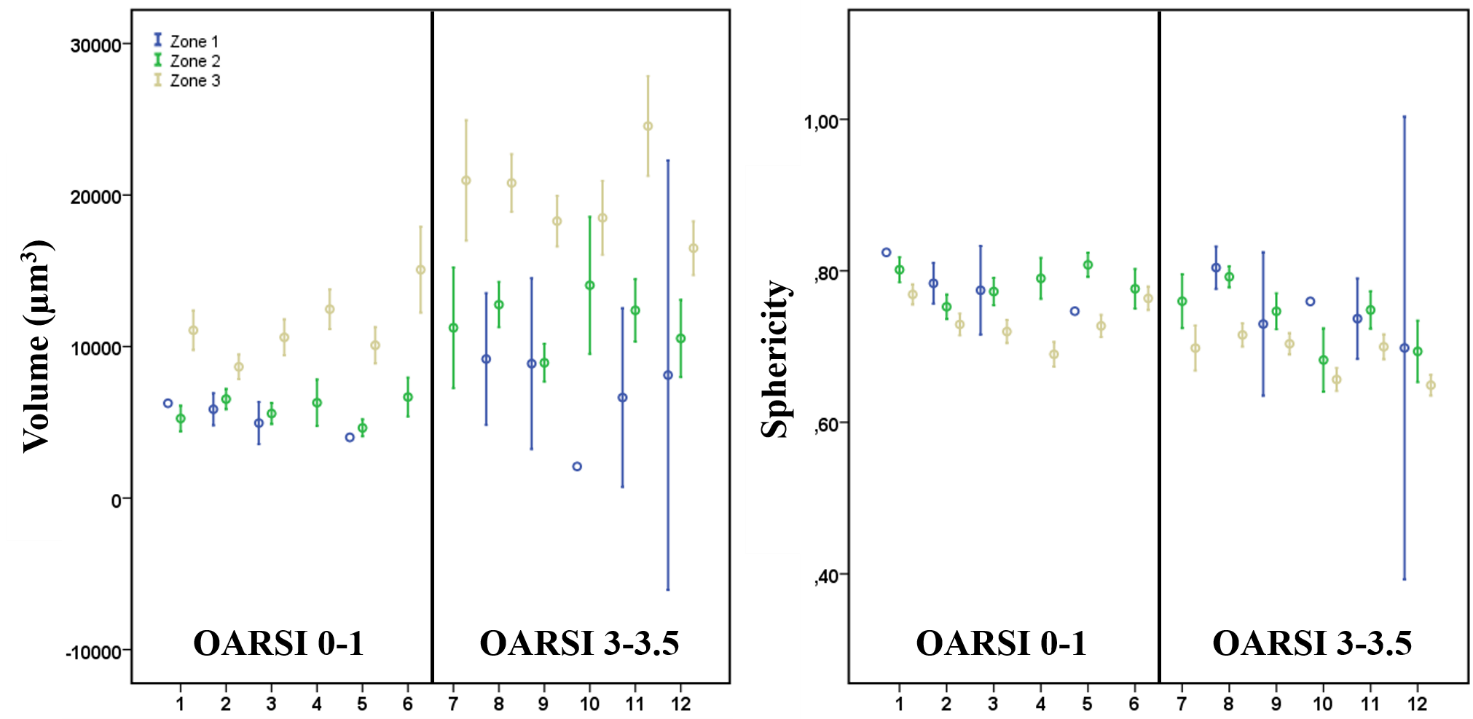


**Figure S6.** The Volume (left) and Sphericity (right) results presented sample-wise: the means with their 95% CIs. As can be seen, the volumes are overall larger, and sphericities smaller in the OARSI grade 3-3.5 samples compared to OARSI grade 0-1 samples.

**HMDS drying µCT –protocol**

This protocol is optimized for 4 mm human osteochondral cores that were cut in half. If implemented to samples with bigger core diameter or to other subjects with thicker cartilage, sample processing times should be higher.

**Drying protocol:**

**Solutions:**

10% Neutral-buffered formalin

30%, 50%, 70%, 80%, 90%, 96%, and 100% Ethanol

Hexamethyldisilazane (HMDS) solution (Sigma: 52619)

**Protocol:**

Volume of the solutions used should be at least 10 times the volume of the tissue to ensure sufficient results.

- Fix the osteochondral core in Formalin for 5 days
- Immerse sample to 30% ethanol at least for 3 hours
- Immerse sample to 50% ethanol at least for 3 hours
- Immerse sample to 70% ethanol at least for 3 hours
- Immerse sample to 80% ethanol at least for 3 hours
- Immerse sample to 90% ethanol at least for 3 hours
- Immerse sample to 96% ethanol at least for 3 hours
- Immerse sample to 100% ethanol at least for 3 hours
- Immerse sample to HMDS solution for 3 hours
- Dry the sample out in air in ventilation cabin in room temperature for at least 8 hours
- Store the sample in an desiccator (or similar container with silicon in container to prevent the sample from moisturizing) while waiting for imaging

**µCT imaging protocol:**

**Sample preparation:**

- Fix the sample to brass sample holder with dental wax and super glue (do not cover the cartilage with anything)
- Leave the sample in the holder (outside the desiccator) for at least 24 hours before imaging in order to let the super glue dry completely therefore preventing the sample from moving during imaging

**Scanning protocol for Skyscan 1272 system:**

- Tube voltage: 40 kV
- Tube current: 250 µA
- Exposure time: 1815 ms
- Additional filtration: No additional filtration
- Binning: 1x1 (projection size: 4032x2688)
- Pixel size: 1.6 µm
- Rotation step: 0.2 degrees
- Averaging frames: 5
- 360° scan
- random movement off

**Sample preservation:**

Samples can be stored out of the desiccator attached in the sample holder for a short period of time (1-2 days) while waiting for imaging; this is even advised in order to minimize the possible movement that the slight re-moisturizing and drying of the super glue might cause. For longer periods of time, remove the sample from the holder and store it in the desiccator.

**References**

1. Thevenot J, Chen J, Finnilä MAJ, Nieminen MT, Lehenkari P, Saarakkala S*, et al*. Local Binary Patterns to Evaluate Trabecular Bone Structure from Micro-CT Data: Application to Studies of Human Osteoarthritis. In: Computer Vision - ECCV 2014 Workshops. L. Agapito, M. Bronstein and C. Rother, Eds. Springer, Cham, 2015, pp: 63-79.
